# Retroperitoneal Fibrosis after Chronic Abscesses of Silicone Fluid Fillers in a Case of Gluteal Augmentation

**DOI:** 10.1155/2020/7236295

**Published:** 2020-06-03

**Authors:** Hana S. Alahmari, Abdurhaman S. Alarfaj, Tariq E. Aljohani

**Affiliations:** ^1^Rheumatology Unit, Department of Medicine, King Khalid University, Abha, Saudi Arabia; ^2^Rheumatology Unit, Department of Medicine, King Saud University, Riyadh, Saudi Arabia; ^3^Department of Pathology and Laboratory Medicine, King Saud University, Riyadh, Saudi Arabia

## Abstract

Filler injection or implantation is a progressing revolutionary subject. Although the widely available kinds in many implications are considered safe, post filler adverse events are not uncommon. These reactions range from mild reactions such as edema or erythema to detrimental reactions such as recurrent infected granuloma or vascular occlusion, which are predominantly related to non-FDA approved materials. Here, we presented a patient with a significant history of gluteal augmentation using unlicensed silicone who developed extensive retroperitoneal fibrosis complicated by deep venous occlusions and obstructive uropathy.

## 1. Introduction

Bovine collagen was introduced to treat wrinkles and soft tissue defects since 1980, and since that time, many products have been developed in the cosmetic dermatology field to smooth wrinkles, treat facial fat atrophy, and provide soft tissue augmentation [1]. There are numerous types of dermal fillers available in the market which can be categorized based on space-filling or stimulatory effects on the dermal microenvironment [2]. Hyaluronic acid gel, collagen gel silicone oil, and polyacrylamide gel are examples of volumizer fillers, while calcium hydroxyl apatite (CHA) and poly-L-lactic acid (PLA) have both volumizing and biostimulatory properties [2]. This revolutionary procedure with its different types and techniques is considered safe with only minor foreign body reactions such as pain, transient swelling, and erythema. However, detrimental adverse effects can also be expected. Namely, chronic infection, soft tissue necrosis necrosis, granulomatous formation, and autoimmune reaction are present [3]. Delayed infections can be attributed to biofilm formation, creating a stubborn ground of multiple resistant microorganisms. Biofilms can interfere with phagocytosis and facilitate microbial resistance, making infection very difficult to be eradicated therefore [4]. Another side effect is filler-related foreign body granuloma which is a chronic inflammatory reaction with various etiologies and can be defined as a tumor composed of a collection of immune cells, mainly macrophages and lymphocytes [5]. Bentkover suggested that foreign body granulomas are caused by granulomatous inflammation after the aggregation of macrophages in response to large foreign bodies that cannot be phagocytosed by macrophages which recruit and activate fibroblasts and, subsequently, a fibrous capsule develops around the material [6]. The overall clinical incidence of foreign body granulomas associated with cosmetic dermal fillers is infrequent and has been reported to range from 0.02% to 1%, depending on the chemical nature of the dermal filler, its surface structure and properties, and the presence of impurities [3, 7]. Moreover, vascular thrombotic events were rarely reported [8, 9] and silicon pneumonitis was reported once [10]. We herein reported a case of retroperitoneal fibrosis developed secondary to the infected silicone material used for gluteal augmentation.

## 2. Case Presentation

**Figure 1:**
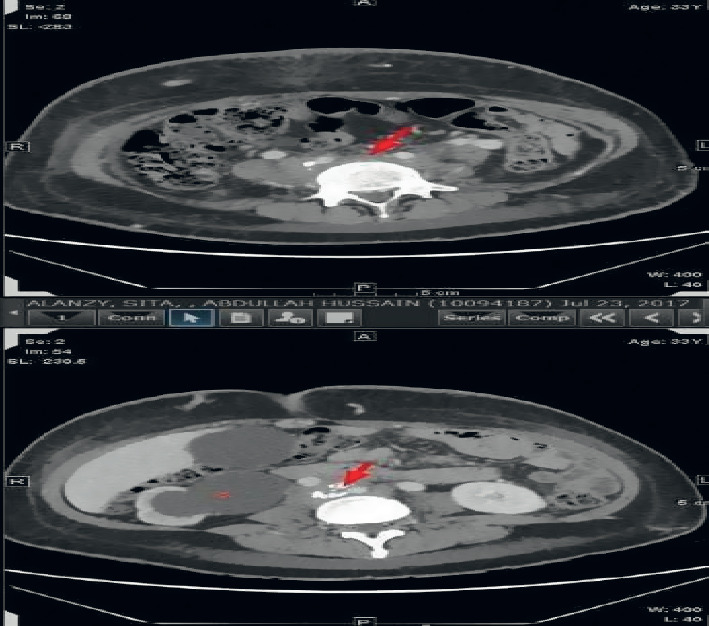
CT abdomen showing retroperitoneal fibrosis (arrow) and right hydroureter (asterisk).

**Figure 2:**
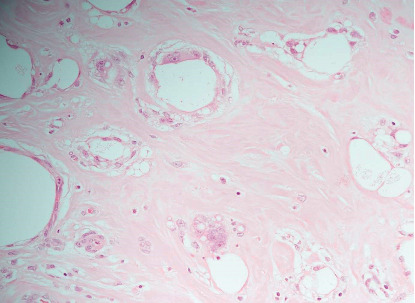
Hematoxylin-Eosin/H&E (200x magnification) staining of the subcutaneous tissue foreign material with fibrosis calcification surrounded by giant cell foreign body reaction and inflammation.

**Figure 3:**
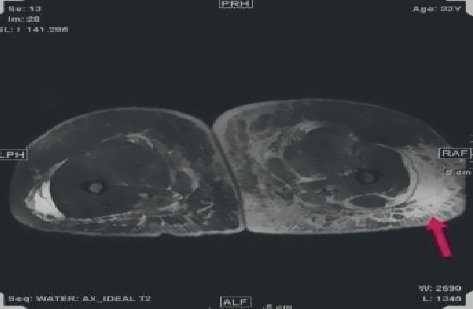
Subcutaneous edema.

A 33-year-old woman presented with a history of permanent silicone injection at the gluteal area 9 years back which was done in a beauty salon by an unlicensed person. In 2016, she underwent partial removal of the filler after a pus discharge from the right gluteal induration burst. The fluid culture was positive for *Pseudomonas aeruginosa* which was treated with intravenous (IV) piperacillin/tazobactam for ten days. Also, she had a history of DVT 3 times in the right proximal leg. The first was 5 years back when she was pregnant at the second trimester, and the second was 2 days after delivery. The third thrombotic event occurred after a few months of filler removal. She presented to the emergency department with marked right leg swelling and pain associated with skin thickness and erythema extended to the lower part of the abdomen. Ultrasound Doppler of the right leg confirmed right iliofemoral DVT. Abdominal CT showed retroperitoneal soft tissue density with multiple calcifications that compress the aorta and inferior vena cava. They also compress the right ureter contributing to hydronephrosis and right proximal hydroureter (Figure 1). No fluid collection had been noticed. In addition, subcutaneous tissue edema and wall thickening of the lower part of the abdominal wall and gluteal area were also reported. She was managed with enoxaparin therapeutic dose and clindamycin and ciprofloxacin for the possibility of cellulitis. Meg 3 demonstrated the nonfunctioning right kidney with only 3% function. A biopsy was taken from the right gluteal area that showed diffuse subcutaneous tissue fibrosis and fat necrosis along with multiple foreign body giant cell reactions (Figure 2). Fungal culture was negative. Serum IgG4 level was normal, and ANA was negative by immunofluorescence and ELISA. Skin-snip biopsy also showed dermal edema with vascular proliferation and chronic inflammatory cell infiltration, representing a reaction to the previous injected material. The retroperitoneal fibrosis was managed with oral prednisolone and methotrexate 10 mg weekly along with folic acid and vitamin D supplements, in addition to warfarin. After the hospital discharge, the right leg was progressively swollen and painful. She experienced multiple times of fluid discharge from the edematous leg over 2 months. She was readmitted as a case of complicated nostra verrucose with possible cellulitis. Venous thrombosis was excluded by Doppler US. MRI of the right leg showed extensive circumferential subcutaneous edema of the right leg and thigh and fat stranding (Figure 3). It also showed multiple collections occupied the medial aspect of the thigh were the largest that measured approximarely 1.4 cm ×  5.7 cm × 9.0 cm in anteroposterior, transverse, and craniocaudal transverse, respectively. Another small collection in the lateral aspect of the lower right limb was 2 × 1.6 cm. Blood culture grew *Streptococcus pyogenes*, and culture from the discharge fluid was positive to *Acinetobacter baumannii*. Intravenous piperacillin-tazobactam 4.5 g was initiated, and methotrexate was paused during hospitalization. After 3 months, she was readmitted for cellulitis and treated with IV pipracilline/tazobactam and clindamycin for 10 days. Finally, one month before writing this report, she was admitted with pyelonephritis. Since her discharge, she is off MTX and prednisolone was gradually tapered as no marked improvement was noticed.

## 3. Discussion

The majority of postfiller reaction cases illustrated in the literature are presenting with painful swelling, nodules, skin hyperpigmentation, and fibrosis [11]. Other known detrimental side effects are infection, filler migration, and granulomatous formation [12]. We address a case of retroperitoneal fibrosis resulting from deep-seated chronic soft tissue infection after gluteal augmentation by injecting silicone. RPF progressed and complicated with vascular occlusion and obstructive uropathy.

RPF is a relatively rare disease that is typically characterized by fibrotic lesions with infiltration by chronic inflammatory cells around the abdominal aorta and common iliac artery and ureter causing obstructive uropathy [13]. It can be idiopathic, which has been considered to be a spectrum of IgG4-elated diseases [14], or secondary to many etiologies such as infection, radiation, drugs, or malignancy.

The recurrent infection after gluteal filler is not common, albeit the prevalence illustrated in the literature represented that cases were operated under medical supervision and none of these cases had developed RPF. The culprit organisms are variable such as Gram-positive and Gram-negative as well as atypical organisms [15]. Propionibacterium acnes had been detected as well [2]. Biofilms are composed of heterogeneous and sessile bacterial colonies supported by a glycocalyx. Once activated, biofilms can cause acute purulent infections and sepsis or chronic inflammation with a subsequent granulomatous response [16]. Biofilms are an important site for the proliferation of microorganisms and antibiotic resistance through creating multiple DNA mutations and that makes it very difficult to grow in culture and very hard to be eradicated due to extended antibiotics resistance [6]. Moreover, cases of postfiller mycobacterium tuberculous abscess were also reported [17, 18]. Another case of nontuberculous mycobacterium infection has been reported after injection of adulterated liquid silicone [15]. Treatment is according to the growing organism and sensitivity, and usually it requires prolonged course of antibiotics.

Surgical resection of the foreign body is a reasonable option. Nevertheless, postsurgical complications such as abscess and fistula are possible especially in cases of foreign body soft tissue granulomatous reaction [19]. In our case, the surgical option was not a successful strategy.

The vascular occlusion can occur through direct intravascular injection of the material or by extraluminal compression of a large volume of fillers [4, 12]. However, venous thrombosis, in this case, was secondary to extensive retroperitoneal fibrosis and warranted lifelong anticoagulant.

Obstructive uropathy was also developed by external compression of the ureters. Ureteral obstruction is usually managed with conservative procedures, such as ureteral stenting or percutaneous nephrostomy [20]. However, both ureteral stents and percutaneous nephrostomy PNS are harmful. Ureteral stents are frequently associated with bladder irritability, hematuria, encrustation, and febrile UTI [20]. However, PNS is associated with catheter-related problems, including infection, obstruction, and poor quality of life [21].

Prognosis of RPF depends on the duration of fibrosis and the presence of the complications such as renal insufficiency and malignancy [22].

## 4. Conclusion

Postfiller complicated infection is not common. However, secondary RPF is a serious medical condition which carries fatal consequences and poor quality of life. Though this condition is very rare, it is important for the practitioners as well as the candidates to be vigilant for such adverse outcomes and avoid injecting permanent or unlicensed materials. The knowledge gap of the underlying pathophysiology and management approach necessitates further prospective work.
